# Interpretation of active-control randomised trials: the case for a new analytical perspective involving averted events

**DOI:** 10.1186/s12874-023-01970-0

**Published:** 2023-06-26

**Authors:** David T. Dunn, Oliver T. Stirrup, Sheena McCormack, David V. Glidden

**Affiliations:** 1 Institute for Global Health, University College London, London, UK; 2 MRC Clinical Trials Unit, University College London, 90 High Holborn, London, WC1V 6LJ UK; 3 Department of Epidemiology and Biostatistics, University of California San Francisco, San Francisco, CA USA

**Keywords:** Active-control, Non-inferiority, Estimand, Averted, Relative efficacy

## Abstract

Active-control trials, where an experimental treatment is compared with an established treatment, are performed when the inclusion of a placebo control group is deemed to be unethical. For time-to-event outcomes, the primary estimand is usually the rate ratio, or the closely-related hazard ratio, comparing the experimental group with the control group. In this article we describe major problems in the interpretation of this estimand, using examples from COVID-19 vaccine and HIV pre-exposure prophylaxis trials. In particular, when the control treatment is highly effective, the rate ratio may indicate that the experimental treatment is clearly statistically inferior even when it is worthwhile from a public health perspective. We argue that it is crucially important to consider *averted* events as well as observed events in the interpretation of active-control trials. An alternative metric that incorporates this information, the averted events ratio, is proposed and exemplified. Its interpretation is simple and conceptually appealing, namely the proportion of events that would be averted by using the experimental treatment rather than the control treatment. The averted events ratio cannot be directly estimated from the active-control trial, and requires an additional assumption about either: (a) the incidence that would have been observed in a hypothetical placebo arm (the counterfactual incidence) or (b) the efficacy of the control treatment (relative to no treatment) that pertained in the active-control trial. Although estimation of these parameters is not straightforward, this must be attempted in order to draw rational inferences. To date, this method has been applied only within HIV prevention research, but has wider applicability to treatment trials and other disease areas.

## Introduction

Active-control trials, in which an experimental treatment is compared with an established treatment, are performed when the inclusion of a placebo control group is deemed to be unethical [1]. For time-to-event outcomes, the primary estimand is usually the rate ratio or the closely-related hazard ratio [2–5]. Here, we present examples which demonstrate that this estimand can be clinically misleading, and highlight the importance of considering *averted* events as well as observed events [6]. We propose an alternative metric which incorporates the number of averted events, thereby avoiding the limitations of the rate ratio. We introduce the problem with a hypothetical COVID-19 vaccine active-control trial.

## Hypothetical COVID-19 vaccine trial

The first licensed COVID-19 vaccine, BNT162b2 (BioNTech/Pfizer), was found to reduce the incidence of COVID-19 by approximately 95% [7]. Imagine that we wished to assess the clinical efficacy of a new COVID-19 vaccine shortly after the licensure of BNT162b2. Given such high clinical efficacy, we conduct a large, active-control trial with 10,000 person-years follow-up per arm, using BNT162b2 as the comparator (Table 1). In this trial we observe 20 cases of COVID-19 in the BNT162b2 arm and 80 cases in the experimental vaccine arm. The rate ratio is very high (4.00, 95% CI 2.42–6.90) – at face value, this suggests that the experimental vaccine is markedly inferior to BNT162b2, arguing strongly against its licensure.

**Table 1:** Results from a hypothetical active-control COVID-19 vaccine trial

	**BNT162b2**	**Experimental vaccine**
Person-years follow-up per arm	**10,000**	**10,000**
Efficacy	**95%**	80%
Observed COVID-19 cases	**20**	**80**
COVID-19 cases if subjects had not been vaccinated	400	400
Averted COVID-19 cases	380	320

Values in bold are known or directly observed, other values are inferred

We now consider a different perspective. The 95% efficacy of BNT162b2 indicates that there would have been 400 (= 20/(1–0.95)) infections in each arm if *none* of the trial participants had been vaccinated. As 80 COVID-19 cases occurred in the experimental arm, this implies that the experimental vaccine averted 320 cases, and that its efficacy was 80% (= 320/400). An efficacy of 80% comfortably exceeds the target of 50% set by the World Health Organization and the US Food and Drug Administration for the licensure for COVID-19 vaccines [8, 9]. Coincidently, 80% is the approximate efficacy of the ChAdOx1 viral-vector vaccine (provided the prime-boost interval is ≥ 12 weeks), which is considerably cheaper than mRNA vaccines and has less stringent cold chain requirements [10]. Thus, if ChAdOx1 had been assessed against BNT162b2 in an active-control trial, the use of the rate ratio could have led to the unwarranted rejection of a viable vaccine option in resource-limited settings. Indeed, ChAdOx1 saved more lives worldwide in 2021 than any other COVID-19 vaccine [11]. An alternative and more meaningful metric than the rate ratio is the efficacy of the experimental vaccine compared with the control vaccine (“relative efficacy”) i.e. 80/95 = 0.842. We return to this metric in Mathematical formulae and alternative approaches to estimating the averted events ratio section.

## Non-inferiority and effect preservation

Active-control trials are often designed and analysed within a non-inferiority framework [2, 5, 12]. A key aspect of non-inferiority trials is the non-inferiority margin, which is pre-specified in the trial protocol, although most trials fail to report a justification for the selected margin [13]. The concept of “preservation of effect” for defining the non-inferiority margin, as recommended in regulatory guidelines, is not widely applied [14]. The underlying idea is that the experimental treatment should demonstrate efficacy greater than a specified fraction of the efficacy of the control treatment. Two key pieces of information are required to use this approach: the efficacy of the control treatment as inferred from previous placebo-controlled trials, and the *fraction* of this effect to be preserved. Conventionally, this fraction has been set at 50%, although it has been argued that higher, more conservative values should be used [3, 15]. Also, for non-continuous outcomes, the *scale* for assessing effect preservation needs to be selected. The standard approach for time-to-event outcomes is to use a log-incidence scale, driven by statistical modelling considerations [3, 4]. However, this scale is arbitrary and inference based upon it lacks clear interpretability, as discussed in the next section.

## HIV pre-exposure prophylaxis trial

HIV pre-exposure prophylaxis is the use of antiretroviral drugs to prevent the acquisition of HIV infection rather than to prevent disease in those already infected with the virus. The first regimen to be approved was the two-drug combination TDF-FTC, which confers very high protection (> 95%) if taken as indicated [16]. DISCOVER was an active-control non-inferiority trial that assessed another two-drug combination, TAF-FTC, against TDF-FTC [17]. Analysis was performed on a log-incidence scale, with the aim of preserving 50% of the effect of TDF-FTC; non-inferiority would be concluded if the upper 97.5% confidence limit for the rate ratio (TAF-FTC versus TDF-FTC) was less than 1.62 [17].

The trial was expected to generate approximately 72 endpoints per arm, but the observed HIV incidence was much lower, with only 11 and 6 incident HIV infections in the TDF-FTC and TAF-FTC arms, respectively (Table 2) [17]. The observed upper 97.5% confidence limit for the rate ratio was 1.48, slightly lower than non-inferiority margin of 1.62, allowing non-inferiority to be concluded. However, this conclusion is very unstable – for example, adding a *single* additional event to the TAF-FTC arm (from 6 to 7) increases the upper 97.5% confidence limit to 1.65 i.e. above the non-inferiority margin. This inferential instability vis-a-vis the observed data is known as a high “fragility index” [18], although the relevance of this concept has been challenged by other researchers [19].

**Table 2:** DISCOVER trial: primary outcome analysis

**Original analysis on rate ratio scale**					
**Group**	**No. subjects**	**PYFU**	**Incident HIV infections**	**Incidence rate (per 100 PYFU)**	**Rate ratio (95% CI)**
TDF/FTC	2693	4386	11	0.251	REF
TAF/FTC	2694	4370	6	0.137	0.55 (0.20, 1.48)
**Re-analysis using an averted infections framework**					
**Group**	**PYFU**	**Observed infections**	**Predicted infections**^**a**^	**Averted infections**	**Averted infections ratio (95% CI)**
TDF/FTC	4386	11	90.4	79.4	REF
TAF/FTC	4370	6	90.0	84.0	1.06 (0.96–1.17)

^a^Applying counterfactual placebo incidence rate of 2.06 per 100 PYFU (lower 2.5% credibility limit)

**Fig. 1:**
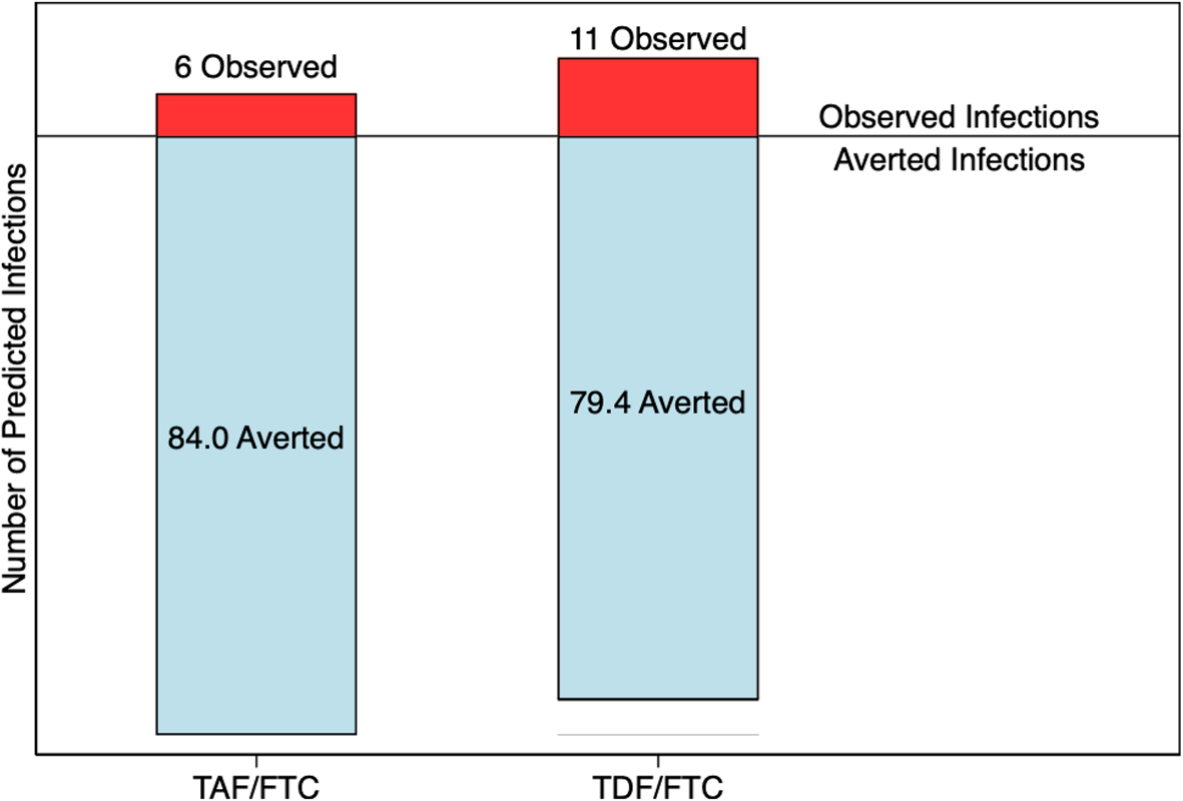
Averted and observed infections in the DISCOVER trial. Assuming counterfactual placebo incidence = 2.06 per 100 PYFU

Glidden and colleagues re-analysed the DISCOVER data using an averted events (infections) framework, based on the counterfactual placebo HIV incidence rate [20]. Using a Bayesian approach that synthesised data on baseline HIV infections and incident sexual transmitted infections, the posterior mean for the counterfactual placebo incidence was estimated to be 4.51 (95% credible interval [CrI] 2.06-7.36) per 100 PYFU. Applying (pessimistically) the lower bound estimate of 2.06 per 100 PYFU gives approximately 90 predicted events in each group, had they received placebo (Table 2). If this value is accurate, both regimens averted substantial numbers of infections: an estimated 79.4 in the TDF-FTC group and 84.0 in the TAF-FTC group (Fig. 1).

An alternative metric to the ratio of the observed events is the ratio of *averted* events (averted events ratio [AER]) between the groups (84.0/79.4 = 1.06, 95% CrI 0.96–1.17). In other words, TAF-FTC prevented an estimated 6% more infections than TDF-FTC, with a plausible range from 4% fewer infections to 17% more infections. With the AER, conclusions about non-inferiority are made on the basis of the lower confidence limit; thus, we can conclude that TAF-FTC preserved at least 96% of the effect of TDF-FTC, emphatically demonstrating non-inferiority. In this framework, adding one extra event to the TAF-FTC arm (decreasing the predicted number of averted infections from 84.0 to 83.0) has no material effect on the averted infections ratio (1.05, 95% CrI 0.95–1.16). When both treatments are highly effective, as here, the AER is much more stable than the rate ratio.

## Alternative estimation approach

Formalising the argument in the previous section, let λ_E_ and λ_C_ denote the observed incidence rates in the experimental and control arms, and let λ_P_ denote the counterfactual placebo incidence. The AER is calculated by

1 $$\Psi = \frac{{\uplambda }_{\mathrm{P}}- {\uplambda }_{\mathrm{E}}}{{\uplambda }_{\mathrm{P}}- {\uplambda }_{\mathrm{C}}}$$

Now let ${\uptheta }_{\mathrm{CP}}= \left({\uplambda }_{\mathrm{P}}-{\uplambda }_{\mathrm{C}}\right) /{\uplambda }_{\mathrm{P}}= {1-\uplambda }_{\mathrm{C}}/{\uplambda }_{\mathrm{P}}$ denote the efficacy of the control treatment (relative to no treatment) and let ${\upbeta }_{\mathrm{EC}}={\uplambda }_{\mathrm{E}}/{\uplambda }_{\mathrm{C}}$ denote the rate ratio (or hazard ratio) observed in the active-control trial. Dividing all terms in Eq. (1) by ${\uplambda }_{\mathrm{P}}$,

2 $$\begin{array}{l}\Psi = \frac{1 - {\uplambda }_{\mathrm{E}}/{\uplambda }_{\mathrm{P}} }{1 - {\uplambda }_{\mathrm{C}}/{\uplambda }_{\mathrm{P}}}\\ = \frac{1 - {{\upbeta }_{\mathrm{EC}}\uplambda }_{\mathrm{C}}/{\uplambda }_{\mathrm{P}} }{{\uptheta }_{\mathrm{CP}}}\\ = \frac{1 - {\upbeta }_{\mathrm{EC}}\left(1-{\uptheta }_{\mathrm{CP}}\right) }{{\uptheta }_{\mathrm{CP}}}\end{array}$$

This formulation reveals that the AER can alternatively be estimated via the counterfactual effectiveness of the active-control treatment, rather than the counterfactual placebo incidence [21]. The choice of which formulation to use depends on the disease context. Because HIV incidence changes relatively gradually in a given population, estimation of the counterfactual placebo incidence may be feasible in HIV prevention research. In contrast, the incidence of SARS-CoV-2 (and thus COVID-19) has fluctuated in a largely unpredictable manner, implying the likely need to perform estimation via the counterfactual vaccine efficacy.

**Fig. 2:**
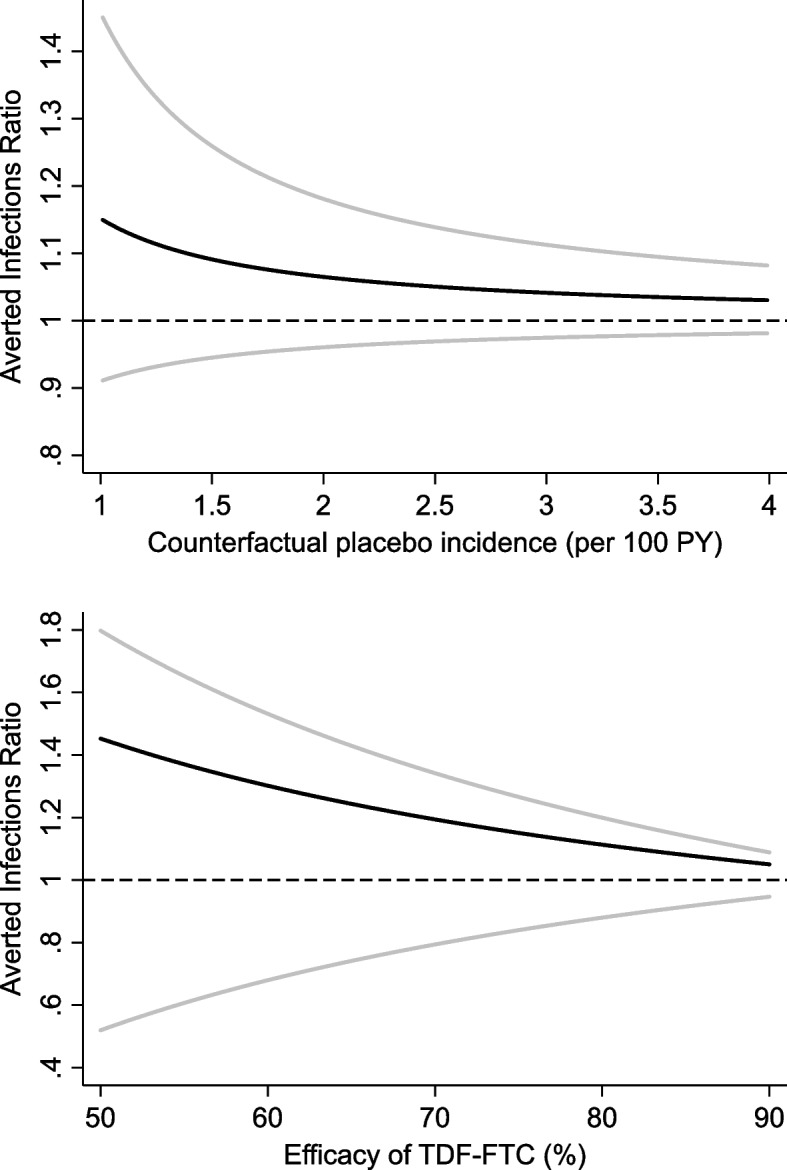
Sensitivity analyses of DISCOVER data. Top panel: Varying counterfactual placebo incidence. Lower panel: Varying counterfactual effectiveness of TDF-FTC. Black line, point estimate. Grey lines, 2.5% and 97.5% confidence limits. Note different scales on graphs. Methods for deriving confidence limits are given in references 21 and 22

Specification of either counterfactual parameter is challenging and requires subject-matter knowledge [6]. A sensitivity analyses of how point estimates and confidence intervals for the AER vary over the range of plausible values is highly informative. Figure 2 depicts such an analysis for the DISCOVER trial, and reveals several important points. First, the lower the value of the counterfactual parameter, the slightly higher the point estimate of the AER. (Conversely, when the experimental treatment is less effective than the control treatment, the AER is less than one.) Second, confidence intervals are considerably narrower at higher value of the counterfactual parameter; thus, for conservative inference low values should be assumed. Third, the confidence intervals are considerably narrower when imputing the counterfactual placebo incidence rather than the counterfactual efficacy of TDF-FTC, favouring the use of the former approach if feasible. Finally, we note that in addition to exploring how the AER varies over a range of values of the counterfactual parameter, we may wish to integrate over this parameter to obtain the unconditional distribution of the AER. Bayesian inference provides a natural framework for this problem [20, 22].

## Relative efficacy

Note that Eq. (2) is simply $\Psi ={\uptheta }_{\mathrm{EP}}/{\uptheta }_{\mathrm{CP}}$ i.e. the efficacy of the experimental treatment compared with the efficacy of the control treatment. This expression may be particularly appealing to vaccinologists, for whom vaccine efficacy is a natural metric. Thus the relative vaccine efficacy estimate of 0.842 in the "Hypothetical COVID-19 vaccine trial" section can be interpreted as the experimental COVID-19 vaccine averting 84.2% of the COVID-19 cases that would otherwise be averted by BNT162b2.

The term relative efficacy or relative effectiveness has been widely used in influenza vaccine research – a recent review paper identified 63 articles that reported this term, either in the comparison of different vaccines, doses of the same vaccine, or vaccination schedules [23]. However, in this context relative vaccine efficacy/effectiveness has been defined as:

3 $$\left({\uplambda }_{\mathrm{C}}-{\uplambda }_{\mathrm{E}}\right) /{\uplambda }_{\mathrm{C}}= 1-{\upbeta }_{\mathrm{EC}}$$

This is interpreted as the proportionate reduction in influenza cases if using the experimental vaccine rather than the control vaccine (without relation to a hypothetical placebo group). While this is a meaningful metric, it is fundamentally different to the way that we have defined relative effectiveness.

A recent modelling paper acknowledged limitations in relative effectiveness, as defined in Eq. (3) [24]. For a fixed value of relative effectiveness, the number of untoward events (hospitalisations) averted was shown to be a function of the absolute efficacy of the control vaccine. These values were computed from the *difference*, rather than the ratio, between the efficacies of the experimental (enhanced) and control vaccines, an equally valid approach. In line with our conclusions, the authors stated: “We showed that relative vaccine efficacy is difficult to interpret when reported without contextual information and on its own is a potentially insufficient metric to measure and compare the benefits of enhanced influenza vaccines” [24].

Relative efficacy is also referred to in FDA guidance on COVID-19 vaccines: “For non-inferiority comparison to a COVID-19 vaccine already proven to be effective, the statistical success criterion should be that the lower bound of the confidence interval around the primary *relative efficacy* point estimate is >-10%” [9]. The guidance document does not explicitly define relative efficacy, but a recent paper on the design of non-inferiority trials for COVID-19 vaccines assumed the definition in Eq. (3) [25]. Clear definition of the term is important to avoid ambiguity.

## Conclusions

We have shown that the standard estimand for analysing active-control trials with time-to-event outcomes, the rate ratio based on observed events, can result in misleading clinical conclusions. Valid interpretation requires consideration of the number of averted events as well as observed events, and the AER provides an intuitive and clinically meaningful measure of the relative effectiveness of the experimental treatment. The AER framework is particularly advantageous when the control treatment is highly effective i.e. the number of averted events greatly exceeds the number of observed events.

In the field of HIV prevention, the need to estimate the counterfactual placebo incidence is increasingly accepted and various approaches have been proposed [6, 26–28]. However, most trials continue to use the rate ratio as the primary estimand, probably due to inherent conservatism in regulatory guidance. Finally, we wish to acknowledge that our work is a development of the work of several authors going back 20 years, whose ideas warrant greater attention [3, 15, 29].

## Data Availability

Not applicable.
